# Simulation of Dielectric Properties of Nanocomposites with Non-Uniform Filler Distribution

**DOI:** 10.3390/polym15071636

**Published:** 2023-03-25

**Authors:** Romeo C. Ciobanu, Radu F. Damian, Cristina M. Schreiner, Mihaela Aradoaei, Alexandru Sover, Ashok M. Raichur

**Affiliations:** 1 Department of Electrical Measurements and Materials, Gheorghe Asachi Technical University, 700050 Iasi, Romania; 2 All Green SRL, 700029 Iasi, Romania; 3 Department of Technology, Technical Faculty, Ansbach University of Applied Sciences, 91522 Ansbach, Germany; 4 Department of Materials Engineering, Indian Institute of Science, Bengaluru 560012, India

**Keywords:** nanocomposites with non-uniform filler distribution, electromagnetic simulation, rendering process of the structures, free-space Nicolson–Ross–Weir procedure for non-symmetrical materials, transmission and reflection parameters

## Abstract

Dielectric properties for nanocomposites with metallic fillers inside a polymer matrix were determined using CST STUDIO SUITE—Electromagnetic field simulation software followed by the free-space Nicolson–Ross–Weir procedure. The structure is randomly generated to simulate the intrinsic non-uniformity of real nanomaterials. Cubic insertions were equated to corresponding spherical particles in order to provide either the same volume index or the same exterior surface index. The energy concentration around the inserts and within the entire material was determined as useful information in practice in order to design materials tailored to avoid exceeding the field/temperature limit values. The paper successfully associated the dialectic measurements with the results from the computer simulations, which are mainly based on energetic effects in electromagnetic applications. The experimental results are comparable with the software simulation in terms of precision. The conclusions outline the practical applications of the method for both electromagnetic shielding and microwave domain/telecommunications applications.

## 1. Introduction

Composites have been widely investigated in recent years in order to discover new technological concepts with better properties, mainly for advanced applications, i.e., electronics, aerospace, or automotive applications. In particular, for electrical applications, insertions of metallic or ceramic micro/nanoparticles into a matrix (usually polymer) are found to provide desirable properties [1,2]. Such investigated composites range from fabrics [2], buildings [3], and construction materials [3], to metallic [4] or polymer [5] matrices or are used in the fabrication of nanoscale metamaterials [6]. At present, many studies address materials for EM interference shielding, one of the main applications among those mentioned above, but there are relatively few studies and simulation models on metallic EM interference shielding nanomaterials [7,8,9,10,11,12]. In all electromagnetic applications, due to the high cost of ingredients, technologies, and test procedures, a preliminary simulation of dielectric properties is required for a better experimental design of technology before effective measurement of dielectric properties of related samples is possible.

Most desirable would be the ability to estimate the required material properties at an initial level in order to plan the nanocomposite behavior. Analytical equations to estimate the dielectric properties of dielectric mixtures exist. For example, some equations in [7] may be adequate candidates: Kraszewski (Equation (1)), Landau, Lifshitz, Looyenga (Equation (2)), and Lichtenecker (Equation (3)), based on the values of the dielectric permittivity *ε_i_* of individual constituents and on the volume fraction of individual components of the mixture, *ν_i_*. However, these equations are based on the macroscopic energetic level and fail to take into consideration the architecture effects of composites at nano/microscale, especially in nanocomposites with non-uniform filler distribution, even if most composites are technologically obtained in such a manner.

(1) $$\sqrt{\varepsilon }=\sum_{i}v_{i}\cdot \sqrt{\varepsilon_{i}}$$

(2) $$\sqrt[{3}]{\varepsilon }=\sum_{i}v_{i}\cdot \sqrt[{3}]{\varepsilon_{i}}$$

(3) $$\ln\varepsilon =\sum_{i}v_{i}\cdot \ln\varepsilon_{i}$$

**Figure 1:**
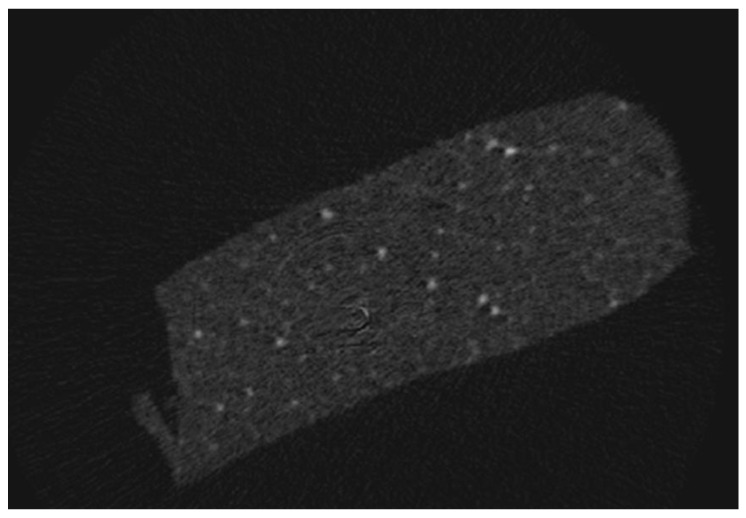
X-ray SKYSCAN 1174 microtomograph (8% ceramic nanofiller in epoxy matrix).

An illustrative example in this direction is presented in Figure 1, in which the investigation made by authors upon an epoxy matrix with 8% ceramic nanofiller was performed using X-ray SKYSCAN 1174 microtomography, and it was emphasized that the insertions are irregular in size and are also irregularly placed inside the matrix even if special technological treatments are used (such as high-speed mixing and/or ultra-sonication to achieve homogeneous dispersion of nanoparticles [13]).

**Figure 2:**
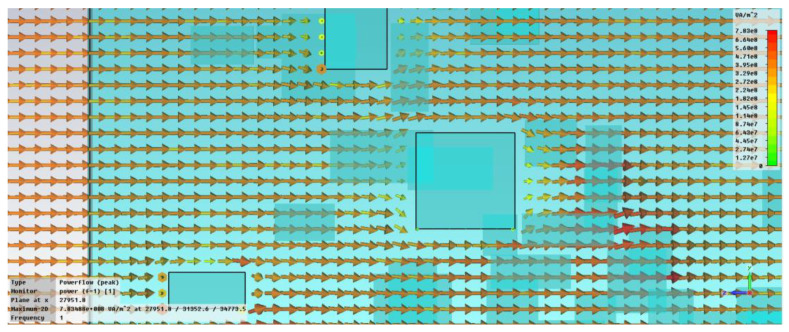
Power flow inside the nanocomposite. Localized shielding and localized absorption.

While the uniform distribution of the filler material has been extensively studied, as in [14], the non-uniform distribution of the nanofiller or uniform distribution of nanofillers with variable dimensions were less addressed. This can generate some specific phenomena at microscale, such as localized increase in absorption features or localized shielding (depending on the type of used nanofiller), shown by authors during the simulation of an electric field as presented in Figure 2. This perturbation of the wave propagation inside the structure will change the macroscopic interaction of the waves within the material, with a clear effect upon the dielectric properties.

Such studies are of great importance for the fabrication of composites with tailored electromagnetic properties, an example being the advanced electromagnetic shielding systems for electronic or automotive applications. Until recently, a very restricted dimension of particles was imposed in order to achieve the desired electromagnetic properties with an imposed very low variation of the particle dimension. Unfortunately, such restrictions put a great pressure on particle suppliers and lead to very increased prices for such tailored particles. Our study will show that such restrictive conditions are not necessary for most applications. Hence, particles with a larger variation of particle dimensions can be successfully used, making the related composites more competitive regarding their price and technology.

## 2. Simulation Method

**Figure 3:**
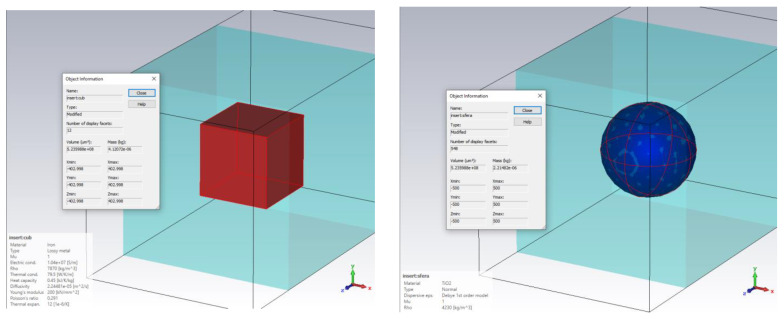

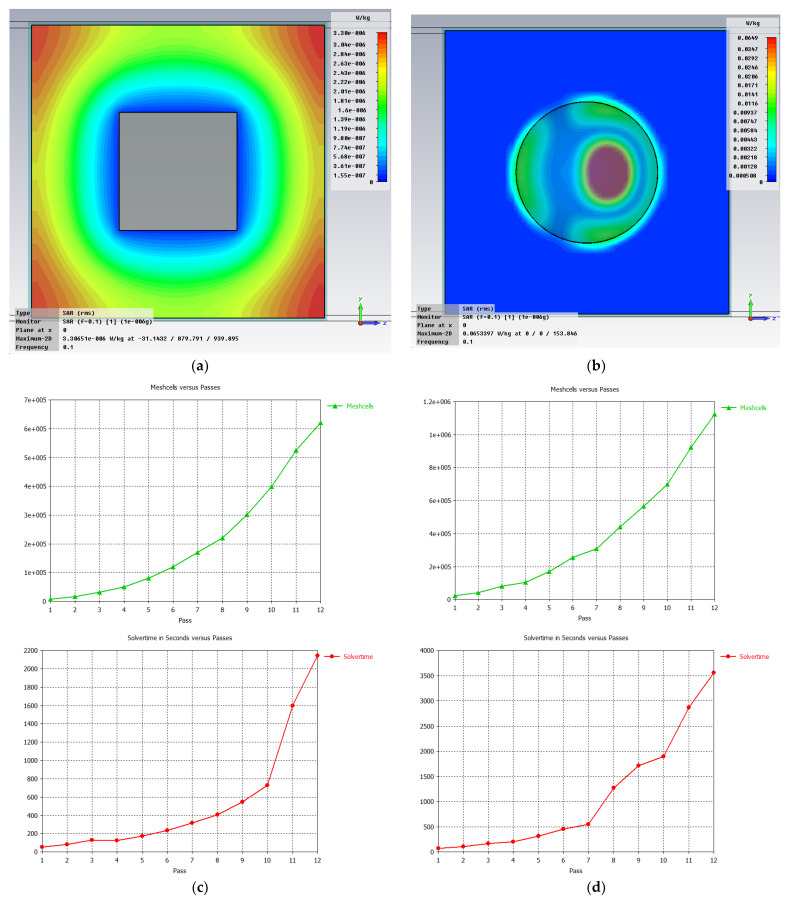
Cubical and spherical particles compared: (**a**) cubical metallic (Fe) particle inside LDPE—simulation conditions and SAR evolution at 0.1 GHz; (**b**) spherical dielectric (TiO_2_) inside LDPE—simulation conditions and SAR evolution at 0.1 GHz; (**c**) number of cells and calculation time required for cubic inserts; (**d**) number of cells and calculation time required for spherical inserts.

In order to properly represent localized effects in simulations, the level of detail of the structure must be increased, going down to the individual particle level. As practically demonstrated, the shapes and dimensions of the individual particles vary widely inside the nanocomposite. As in many software simulations programs, round or spherical shapes are harder to mesh and compute; such simulations lead either to small mesh steps or to memory and computing time requirements that are too great, as in Figure 3.

In a demonstrative preliminary simulation, we used an adapted model derived from CST STUDIO SUITE—Electromagnetic field simulation software [15]. In order to check if a rectangular model for a simulated particle is accurate enough, we investigated the difference between the two geometric models (Figure 3). Both metallic (Fe and/or Al) and lossy dielectric (TiO_2_ and/or Al_2_O_3_) individual particles included within a LDPE—low density polyethylene—matrix have been considered.

**Figure 4:**
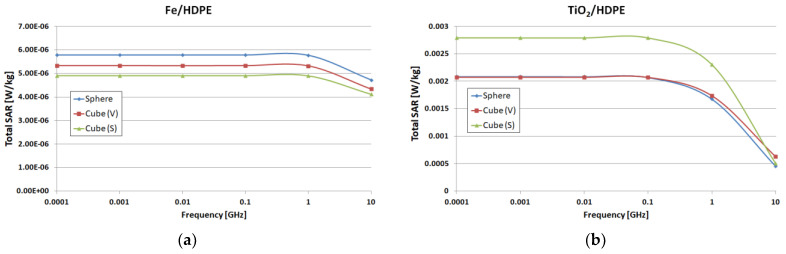
Total SAR (W/kg) results for spherical particles for the equivalent cubical particles (same volume—V) and equivalent cubical particles (same surface—S), respectively: (**a**) metallic (Fe) particle inside HDPE; (**b**) dielectric (TiO_2_) particle inside HDPE.

The cubical particle had the edge computed in the way that the particle had either the same volume ($V=R_{V}^{3}=4\pi \cdot R_{sph}^{3}/3;\;R_{V}=R_{sph}\cdot \sqrt[{3}]{4\pi /3}$, index V in Figure 4) or the same exterior surface ($S=6\cdot R_{S}^{2}=4\pi \cdot R_{sph}^{2}\to R_{S}=R_{sph}\cdot \sqrt{2\pi /3}$, index S in Figure 4), similarly to the corresponding spherical particle. This allows comparison of both volume absorption (in the case of the lossy dielectrics) and surface absorption/shielding (mostly for lossy metals). We computed and compared total SAR (specific absorption rate) as defined in [16] (averaged on a much smaller mass instead of 1/10 g). Results as shown in Figure 4 show that a rectangular/cubical model accurately describes the interaction of the wave with the particle if we keep the same volume (even in the case of the metallic particles, for which we expect predominant surface losses).

In industrial processes, the mix of materials is made by using mass measurements, so the defining recipe for a composite is the mass ratio (MR) of the filler within the composite. However, for tailoring the model in CST, a geometric transformation process is essentially needed. Thus, the volume becomes the most important parameter, so the nanocomposite can be more adequately described by the volume ratio (VR) of the filler in the composite. In [17], the density of the composite is computed (Equation (4)), so we can compute the volume ratio (VR) corresponding to a specific mass ratio (MR) if we know the density of the polymer matrix *ρ_m_* and the density of the filler *ρ_f_* (Equation (5)).

(4) $$\rho_{T}=\left(1-VR\right)\cdot \rho_{m}+VR\cdot \rho_{f}$$

(5) $$VR=\frac{MR\cdot \rho_{m}}{\left(1-MR\right)\cdot \rho_{f}+MR\cdot \rho_{m}}$$

For the specific structures we investigated (7% Al and Fe powders, respectively in LDPE) using LDPE: *ρ_m_* = 0.922 g/cm^3^; Fe: *ρ_f_* = 7.87 g/cm^3^; Al: *ρ_f_* = 2.7 g/cm^3^; we obtain Fe(7%): *VR* = 1.008% and Al(7%): *VR* = 2.884%. The edge of the reference cubic particle with the same volume as the ideal spherical particle will be R_ref_ = *R*∙(4π/3)^1/3^. Two dimensional types of particles have been used in simulation and in further real experiments: 50 nm and 800 nm. Thus, the edge of the cubical reference particle will be *R_ref_*: 80.6/1289.59 nm.

To account for the variability in size of dimension and size of the particles (Figure 1), we used the CST Visual Basic macro interface to draw the particles, and we represent each individual filler particle as a rectangular solid with a random position inside the polymer matrix and length, width, and height randomly obtained as in Equation (6):

(6) $$L_{i},W_{i},H_{i}=R_{ref}\cdot 2^{2x-1}$$

(7) $$V_{i}\in \left(\frac{1}{8}\cdot V_{ref};8\cdot V_{ref}\right)$$

For a generated random number *x* between 0 and 1 (in Equation (6)), every dimension will be at minimum half and at maximum twice the reference cubical edge for 50/800 nm. Thus, the individual volume of the generated particle will be between 1/8th and 8 times the volume of the reference 50/800 nm sphere.

For uniform particles—as described in, e.g., [14]—the number of individual particles required to reach a specific VR can be found (Equation (8)) if we know the dimensions of the matrix:

(8) $$VR=\frac{N\cdot R_{ref}{}^{3}}{L\cdot W\cdot H}$$

However, with random distribution of the individual volumes of the filler particles (Equation (7)), we cannot use Equation (8) to compute the exact number of particles. Instead, we will keep track of the total filler volume by summation Σ *V_i_* inside a “while loop”, and we will stop the procedure when the required VR has been reached. We can compute an estimated number of particles with Equation (8), but the actual number of particles will vary between subsequent runs.

**Figure 5:**
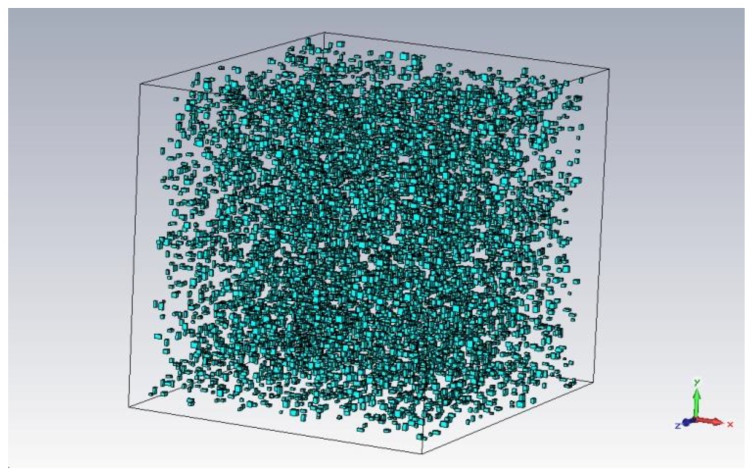
Simulation model with maximum number of random particles (8000).

**Figure 6:**
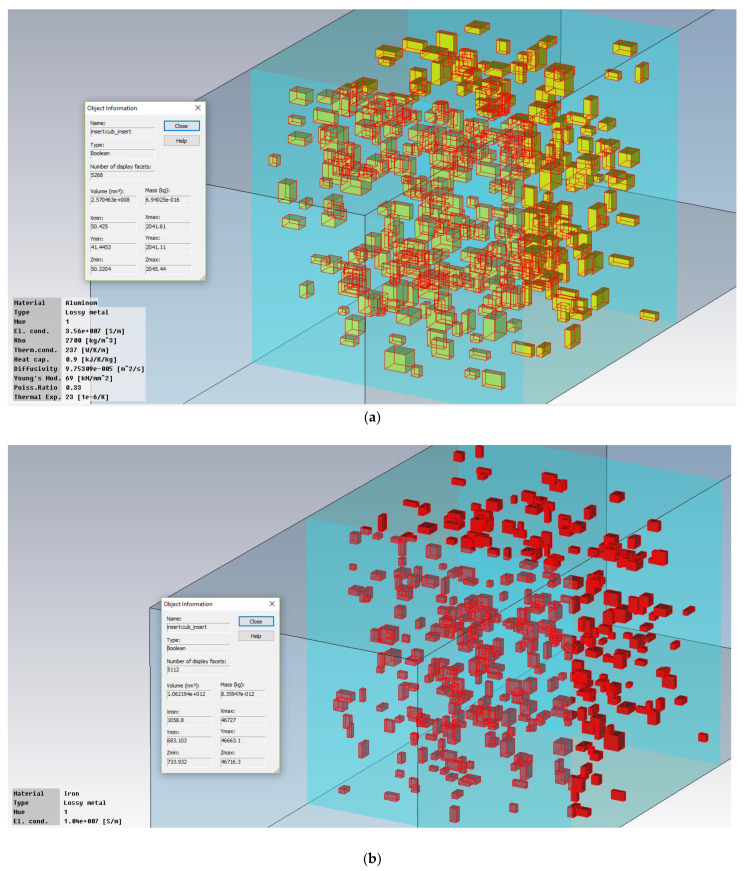
Simulated structures: (**a**) Al (7%) 50 nm, 394 particles, VR = 2.8048% and (**b**) Fe (7%) 800 nm, 405 particles, VR = 1.00024%.

The maximum number of particles that could be generated was about 8000 (Figure 5). However, this number was found to be impractical. The rendering of the 3D model took 12 h, and subsequent analyses could not be performed. Therefore, we chose *n* = 500 particles, and we used Equation (8) to compute the required (cubic) dimensions for such a matrix. Two examples of structures on which simulations were performed are presented in Figure 6.

Because the rendering process of the structure is basically a random process, we can expect a variance in the properties of the structure (VR) and also in the results. Total SAR computed for 20 consecutive runs is plotted in Figure 6—e.g., for TiO_2_ (10%), target VR = 2.365%. The mean VR is 2.333%, with actual values between 2.313% and 2.383%.

**Figure 7:**
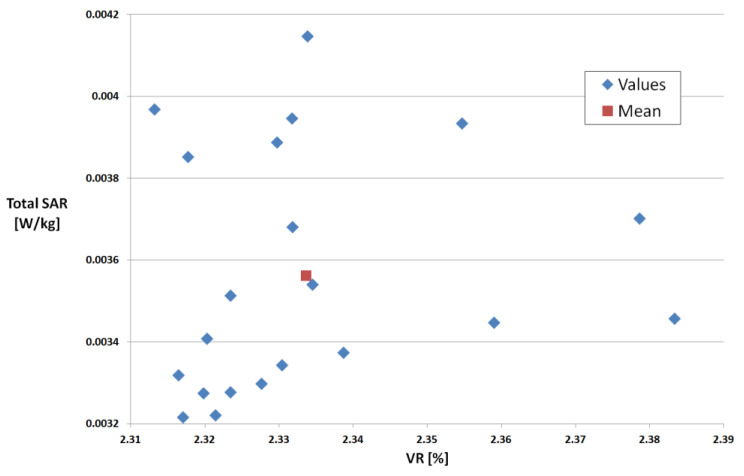
Results for 20 consecutive runs, TiO_2_ (10%), target VR = 2.365%.

The results as presented in Figure 7 are practically unifying the particles’ dimension towards an average volume index V, and towards an average exterior surface index S, so the restrictive conditions of particles dimension may not be necessary for most of the electromagnetic applications, making the related composites more competitive regarding their price and technology.

However, the major importance of the method presented above is related to the practical procedure for equating quadratic particles with spherical particles in electromagnetic analysis in order to properly characterize the important composites with metallic fillers—extensively used in electromagnetic shielding applications—because the commercial software operates with spherical models for particles analysis and metallic particles are mostly of cubical/prismatic shape.

The analysis of field results provides local information on the electromagnetic energy phenomena involved. The integration of these results over the entire analyzed volume provides a characterization of the entire material at the macroscopic level. The most effective parameter for characterizing the electromagnetic effect upon matter is SAR—specific absorption rate—the unit of measurement for the amount of radio frequency energy absorbed in matter. The SAR calculator normally requires the integration of the electric field (V/m), conductivity of the material (S/m), and mass density (kg/m^3^), and SAR can be formulated as the absorbed power density with value in W/Kg. CST software can perform such integrations, calculating the average specific absorption rate in the material (Total SAR (W/kg)), the maximum local specific absorption rate (max. point SAR (W/kg)), and respectively the average absorbed power density (W/m^3^).

As previously mentioned, in order to minimize the effect introduced by the modification of the dimensions of the structure as much as possible, depending on the dimensions of the insertions, a reference parallel analysis is performed in each case considering the matrix without insertions. Finally, from the obtained data, the increase in the average specific absorption rate and the average absorbed power density is calculated and compared to this reference analysis. A max SAR/total SAR ratio is also calculated from the values obtained via CST as an indication of the energy concentration around the inserts, a useful piece of information in practice when designing materials tailored to avoid exceeding the field/temperature limit values (to avoid piercing/melting in case of extreme electromagnetic power levels).

**Figure 8:**
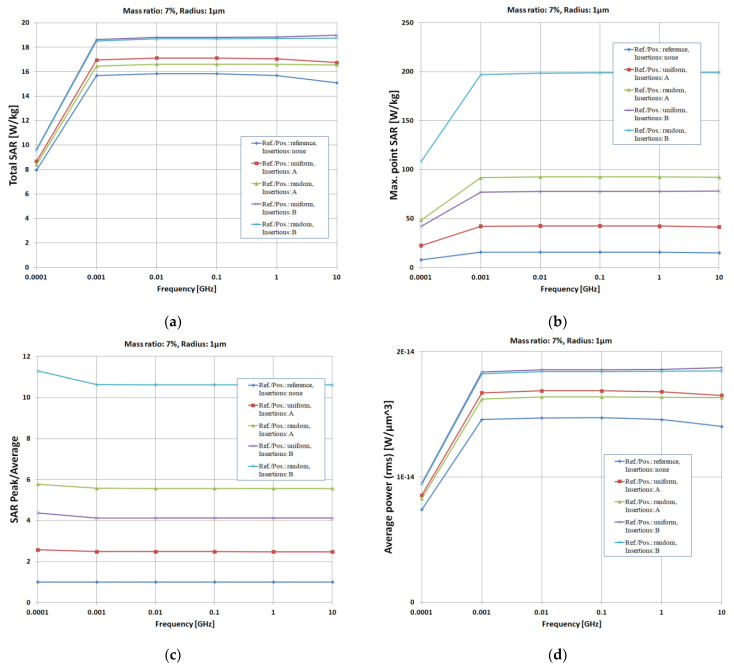

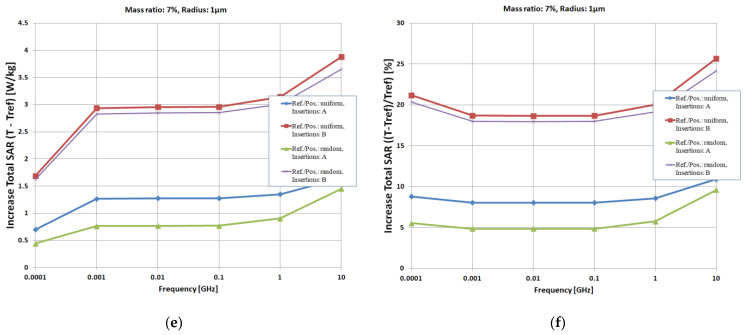
Simulated effect of insertions (type/positioning), MR = 7%, equivalent radius 1 μm: (**a**) - Total SAR (W/kg); (**b**) – Max. SAR (W/kg): (**c**) – SAR peak; (**d**) – Average power (W/μm^3^); (**e**) – Increase of Total SAR (W/kg); (**f**) – Relative Increase of Total SAR (%).

The simulations presented below in Figure 8 are for 7% insertions of TiO_2_ (A type in Figure 8) and Fe (B type in Figure 8), both with 800 nm dimension (equivalent to a model of 1 μm equivalent radius).

The beneficial effect of the insertions on the electromagnetic absorption capacity of the substrate material can be observed. An increase of up to 80% of the average dissipated power is noticed. The material used in insertions is of particular importance, as the particles leading to composites with the highest permittivity and with the highest dielectric losses are recommended. In the analyzed cases, type-B insertions—metallic (leading to composites with ε_r_ = 12, tan δ = 0.09)—present a practically doubled shielding effectiveness compared to type-A insertions—ceramic (leading to composites with ε_r_ = 7, tan δ = 0.05)—for any particle size.

Consequently, ab initio simulation of dielectric properties of nanocomposites with non-uniform filler distribution is of great importance in practice, and the following description presents a method by which the dielectric measurements in industrial practice can be associated with the results from the computer simulations. Here, the key issue is to identify the best way to compute dielectric properties from the parameters determined as presented above through the use of CST Studio Suite - Simulation and analysis of electromagnetic fields, [15].

## 3. Comparative Results

For the purpose of the paper, i.e., to investigate the effect of non-uniform fillers distribution, a variable percent of filler is not needed in either the experimental or simulation procedure. According to the literature, for such composites, a percentage of over 7% (but preferably under 10% due to optimal performance vs. cost balance) is enough for a relevant study. In our case, the study was based on 7% filler concentration but with two different particle sizes at nanoscale, which better models the purpose of the paper (dimensional difference of particles of 1 to 10). Hence, both the original experimental and simulation results are related to such composites, which is not mentioned previously in the literature—i.e., moldable or 3D printable electromagnetic shielding composites with LDPE as polymer matrix and with Al and Fe nanofillers with two different particle size at nanoscale, i.e., 50 nm and 800 nm reference dimensions of the particles.

**Figure 9:**
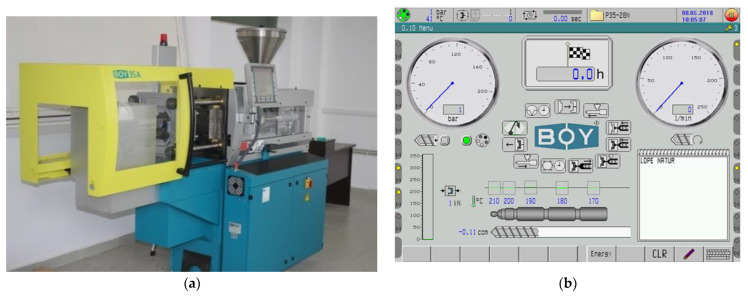
The injection machine (**a**); the control monitor of the injection machine (**b**).

In order to manufacture the samples for the proposed tests, the polymer and the nano-conductive powder were homogenized by mixing for one hour in a TURBULA T2F cylindrical mixer (ARTISAN TECHNOLOGY GROUP, Champaign, IL, USA), with a 1.3 L capacity mixing basket and a rubber-ring-based clamping device with a rotation speed of 40 rpm. Furthermore, the composite materials were obtained on the injection machine Dr. Boy 35A (Dr. Boy GmbH & Co. K, Neustadt-Fernthal, Germany) (screw diameter 28 mm, L/D ratio 18.6 mm, calculated injection capacity 58.5 cm^3^, maximum material pressure 2200 bar, real injection capacity min 500 mm), Figure 9a. For all the experimental models, a 3% percentage of compatibilizing agents was used (a polyethylene graft copolymer with maleic anhydride—PEgMA in a proportion of 1%, poly(ethylene-co-acrylic acid—EAA copolymer in a proportion of 1%, and Tegomer^®^ E 525—amphiphilic copolymer introduced in a proportion of 1%). The optimal temperature regime of the injection process related to the five heating zones of the injection cylinder are indicated in Table 1 and also can be visualized on the control monitor of the injection machine, Figure 9b.

**Figure 10:**
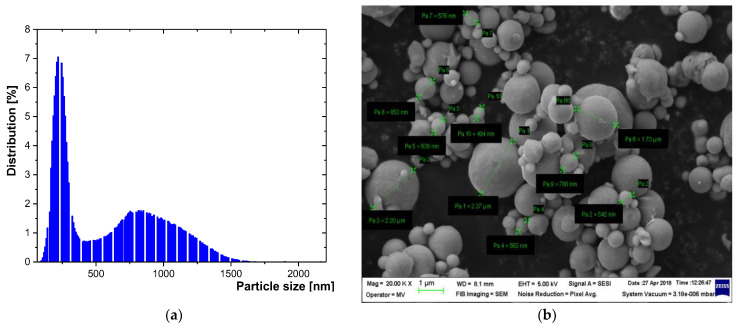
(**a**) Histogram—particle size distribution for Al/800 nm powder; (**b**) SEM image of Al/800 nm powder.

**Figure 11:**
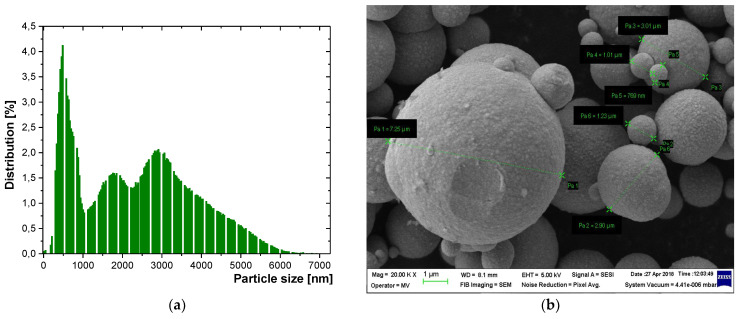
(**a**) Histogram—particle size distribution for Fe/800 nm powder (**b**) SEM image of Fe/800 nm powder.

The features of Fe and Al powders are presented in Figure 10 and Figure 11, which clearly emphasize the fact that, in the technological experiments, the theoretical assumptions of the CST simulation procedure presented above are in concordance with the un-homogenous dimension of real powders and that the dielectric evaluation of such materials must follow the respective model.

**Figure 12:**
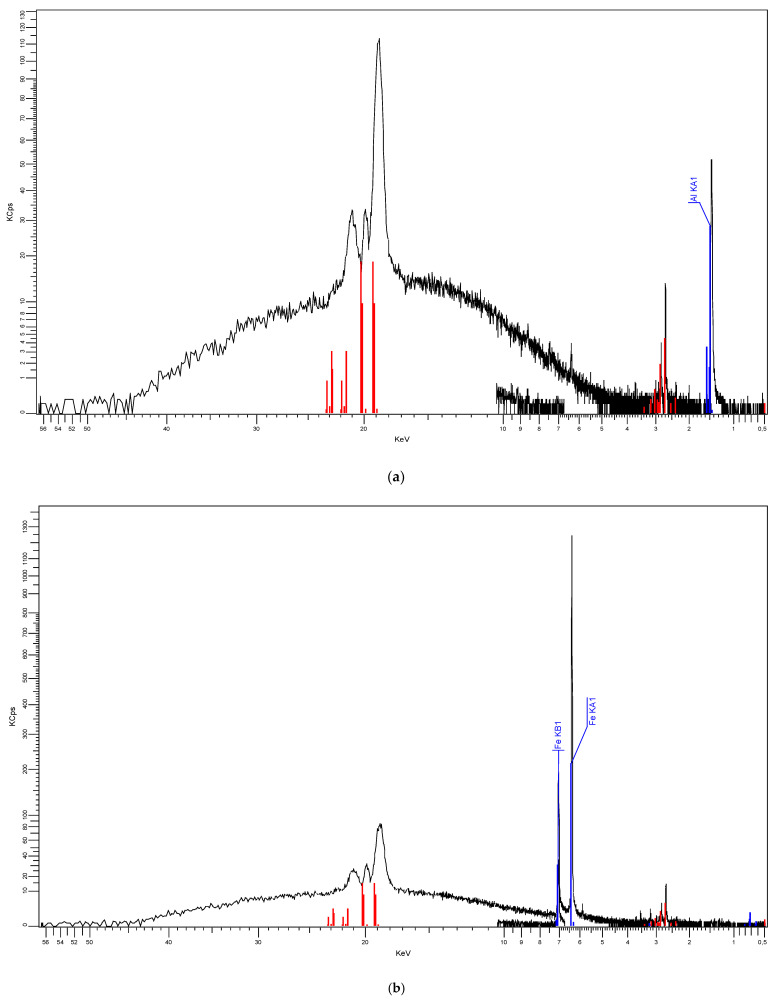
XRF characteristics, 7% mass ratio; (**a**) Al fillers in LDPE matrix, (**b**) Fe fillers in LDPE matrix.

The results obtained as XRF characteristics, Figure 12, confirm the nature and percentages of nano-powder in composite materials.

**Figure 13:**
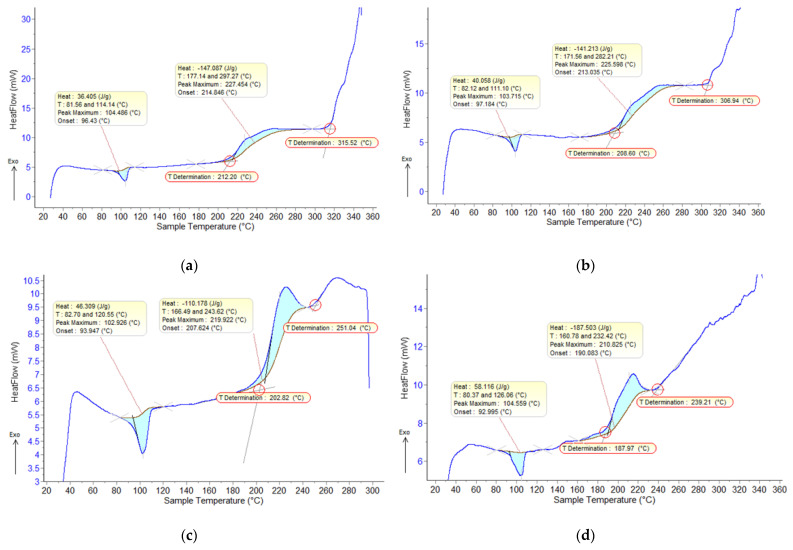
DSC characteristics (air, 10 °C/min), 7% mass ratio; (**a**,**b**) Al fillers in LDPE matrix—800 nm and 50 nm; (**c**,**d**) Fe fillers in LDPE matrix—800 nm and 50 nm.

From the statistical interpretation of the results of the DSC characteristics (air, 10 °C/min), Figure 13, it was found that all the melting temperatures are very close due to the majority concentration of the base polymer. The oxidation process takes place in two stages with different temperature values. The temperatures of the start of the first oxidation process vary in the range of 190–215 °C, and the temperatures of the start of the second oxidation process vary in the range of 250–315 °C. The dimension of the fillers has low influence on thermal processes, but the composites with Fe particles present greater oxidation compared to the ones with Al particles.

### 3.1. Broadband Dielectric Measurements

**Figure 14:**
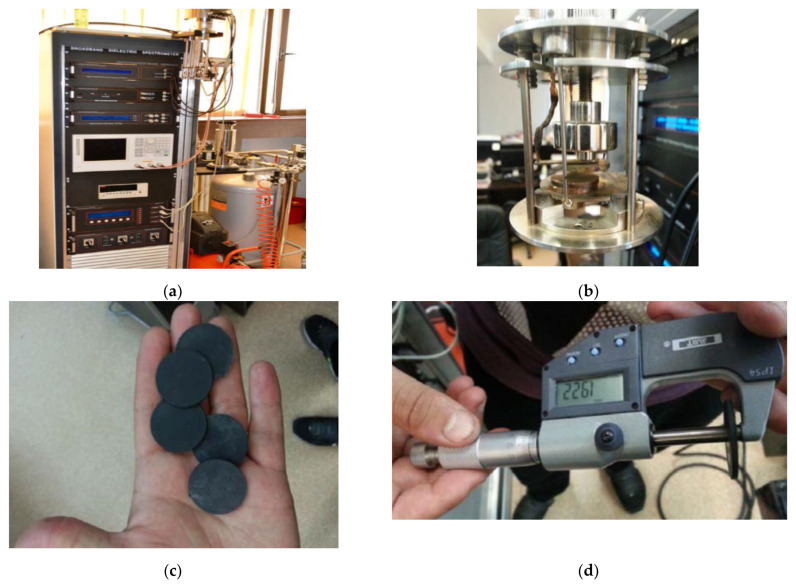
Measurement setup; (**a**) broadband dielectric spectrometer, (**b**) measurement cell, (**c**) manufactured samples, (**d**) measurement of individual sample thickness.

Measurements were performed upon the composite samples obtained in the laboratory with the type of nanofillers mentioned before, Figure 14. Measurements (including those for the LDPE matrix only) were carried out using the Broadband Dielectric Spectrometer (Novocontrol GMBH, Montabaur, Germany) encompassing an alpha frequency response analyzer and Quattro temperature controller (Figure 14a) with tailored measurement cells (as in Figure 14b). The manufactured samples (7% mass ratio Al and Fe inside a LDPE matrix; 50 nm and 800 nm, respectively, Figure 14c,d) were sandwiched between two copper electrodes of 20 mm diameter and placed inside the temperature-controlled cell (Figure 14b).

**Figure 15:**
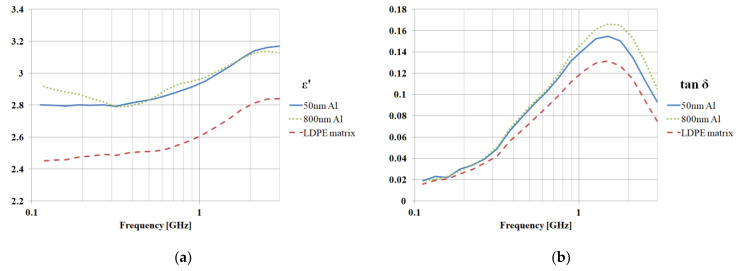

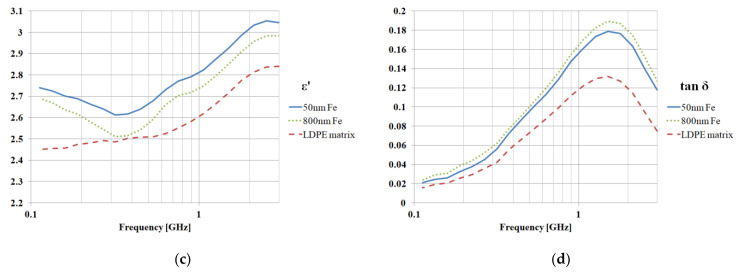
Measurement results, dielectric properties, permittivity, and loss angle tangent, 7% mass ratio; (**a**,**b**) Al fillers in LDPE matrix; (**c**,**d**) Fe fillers in LDPE matrix.

Measurement results are plotted in Figure 15. The use of aluminum nanofillers increases the dielectric permittivity by 13%, while the loss tangent increases by around 20%. Iron nanofillers have an increased effect on losses—the increase in permittivity is around 6%, while losses increase by as much as 40%.

### 3.2. Simulations of Dielectric Parameters and Comparison with Measurements

The free-space Nicolson–Ross–Weir procedure (NRW) considers an infinite planar sample of thickness *d* placed under the illumination of a plane wave [18,19,20]. While this setup is difficult to implement accurately for measurements, in the case of the computer simulation, the structure definition and numerical computation are simpler than other simulation setups. The method has been used [21] to determine electromagnetic properties of engineered materials. In [18,19], the computation steps to determine the complex permittivity of a nonmagnetic sample from the transmission and reflection parameters (S_11_ and S_21_) are detailed.

(9) $$\varepsilon^{*}=\varepsilon^{\prime }-j\cdot \varepsilon^{\prime \prime }=\varepsilon^{\prime }\cdot \left(1-j\cdot \tan\delta \right)$$

(10) $$K=\frac{S_{11}^{2}-S_{21}^{2}+1}{2\cdot S_{11}}$$

(11) $$\Gamma =K\pm \sqrt{K^{2}-1}$$

(12) $$T=\frac{S_{11}+S_{21}-\Gamma }{1-\left(S_{11}+S_{21}\right)\cdot \Gamma }$$

(13) $$\gamma =\frac{\ln\left(1/T\right)}{d}$$

(14) $$\varepsilon^{*}=\frac{\gamma }{\gamma_{0}}\cdot \left(\frac{1-\Gamma }{1+\Gamma }\right)$$

Equations (9)–(14) allow us to compute dielectric properties from the *S* parameters, where *ε* represents the dielectric permittivity with its complex value ε*; tan*δ* is the dielectric loss tangent; *K* is an intermediary variable; Γ and *T* are the transmission and reflection coefficients, respectively; *γ* is a calculated parameter for the electric load of the model; and *d* is the sample thickness.

**Figure 16:**
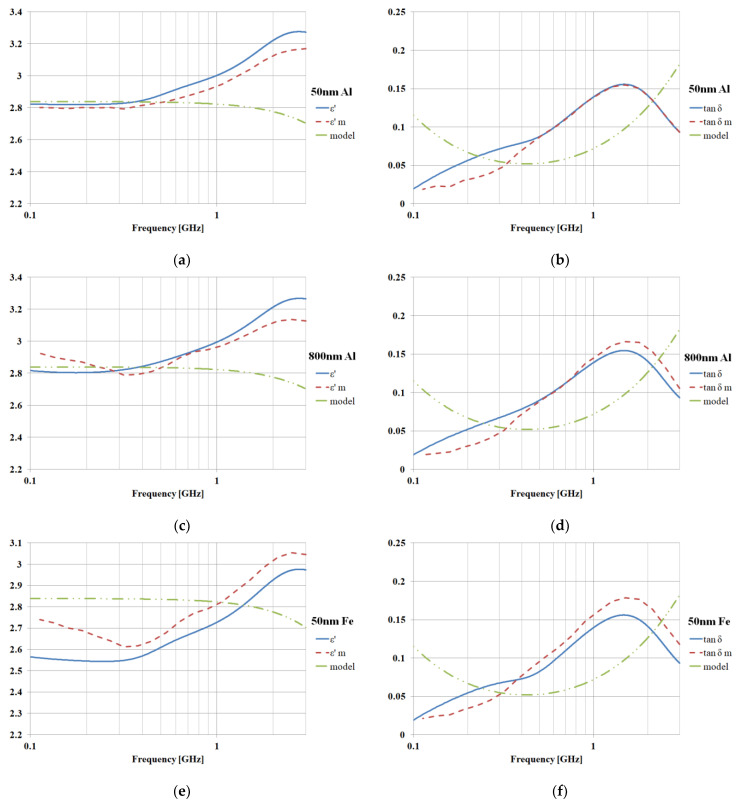

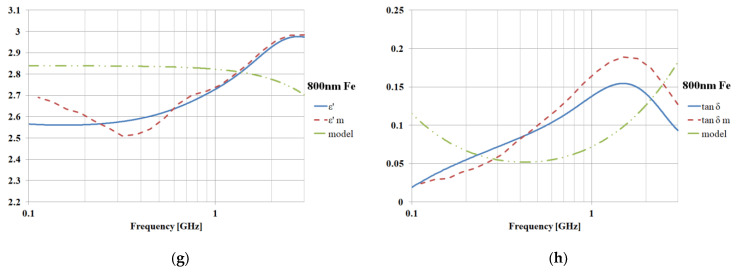
Dielectric properties, comparison with measurement (index m for measured parameters) and with LDPE matrix model; (**a**,**b**) Al 7% 50 nm, (**c**,**d**) Al 7% 800 nm, (**e**,**f**) Fe 7% 50 nm, (**g**,**h**) Fe 7% 800 nm.

The model for the LDPE matrix in CST simulations will be a dispersive material based on measurements performed on actual real samples (Figure 16, Table 2). The CST matching algorithm offers a second order dispersive model, as it offers the closest match in the measurement range (0.1–3 GHz). The frequency-dependent dielectric properties are determined as in Equation (15), a second-order Debye model being a superposition of two different first-order models sharing the same high frequency limit [22].

(15) $$\varepsilon^{*}\left(\omega \right)=\varepsilon_{\infty }+\frac{\varepsilon_{S}-\varepsilon_{\infty }}{1+j\cdot \omega \cdot \tau }$$

where *ε_S_* and *ε*_∞_ are the static dielectric constant and the optic region permittivity, respectively.

Because in CST we cannot place the ports near dispersive materials, we must provide a free space of equal thickness to the sample before the composite. In [18], we find Equation (16) to be required for the de-embedding to be applied to the *S* parameters. The same reference [23,24] suggests the correction in Equation (17) to be applied to *S* parameters before using the free-space Nicolson–Ross–Weir procedure in the case of non-symmetrical samples (and a random distribution of particles will obviously lack the symmetry). We consider the normalized electric fields in the specimen region (0, *L*) for a coaxial line with a matched *γ* load.

(16) $${S^{\prime }}_{11}=S_{11}\cdot {\left(e^{\gamma_{0}\cdot L_{1}}\right)}^{2};\;{S^{\prime }}_{21}=S_{21}\cdot \left(e^{\gamma_{0}\cdot L_{1}}\right)\cdot \left(e^{\gamma_{0}\cdot L_{2}}\right)$$

(17) $${S^{\prime }}_{11}=\frac{S_{11}+S_{22}}{2};{S^{\prime }}_{21}=\frac{S_{21}+S_{12}}{2}$$

The following simulations based on the above theoretical considerations were performed using CST Microwave Studio as well. A dispersive model (second order) was used for the LDPE matrix and the standard (CST library) models for the metallic fillers. The boundary conditions were set to electric wall (x direction walls) and magnetic wall (y direction walls) in order to force the plane wave symmetry for the fields inside the structure [25]. Two Visual Basic (VBA) scripts were used to compute (Equation (8)) the required dimensions of the LDPE matrix and then to create random (position and size) rectangular particles inside, the final structure being similar to those in Figure 5. Upon saving the project, CST keeps a record of the generated random insertions so subsequent opening of the same project will not trigger a new random generation. However, a second, reset script was required to erase all generated structures in order to use the generation script again (for multiple analyses to obtain results, as in Figure 6). We used a tetrahedral mesh with at least eight steps of adaptive tetrahedral mesh refinement in a convergence process. The required amount of RAM for the simulation was about 4GB. The Frequency Domain Solver was used to obtain the complex S parameters, which were then fed to a Nicolson–Ross–Weir procedure (Equations (9)–(14), (16), and (17) to obtain results in Figure 15, in which we compare the simulated and measured dielectric properties. We also plot the dielectric properties of the LDPE matrix (second-order model), and it is obvious that the final results will depend less on the parameters of the matrix model and more on the nanofiller structure. Good agreement between the measured and computed properties is obtained, so we can conclude that the relative variation of the dielectric properties is controlled by the non-uniform nanofillers rather by the polymer matrix. We can recover the relative-frequency-dependent variation of the dielectric parameters even when using an imperfect (but accepted in CST) matrix model. While the absolute values of the dielectric permittivity and loss angle tangent are dependent on the matrix, the behavior in specific frequency ranges is clearly filler-dependent.

The average value of the difference between the experimental values (ε, tanδ) and simulated values (ε—m, tanδ—m) does not exceed 10%, a precision fully accepted in electrical engineering practice, where a tolerance of 20% is normally considered a limit in dielectric parameter estimation.

An additional advantage of the method presented in the paper is the extension of the measurement range for dielectric test equipment, e.g., the Novocontrol Broadband Dielectric Spectrometer (one of the most performant equipment of market) normally has a frequency range of 0.1–3 GHz. However, if we are interested in characterizing a material outside this frequency range, we can use measured parameters to obtain a model valid in the 0.1–3 GHz range and then extend it via simulation toward higher frequencies. The error compared to normal dielectric measurements is less than 5%, and we can obtain valid information in surrounding frequency ranges, for which the normal measurements may involve equipment with prohibitive price unjustifiable as an investment for occasional niche applications.

**Figure 17:**
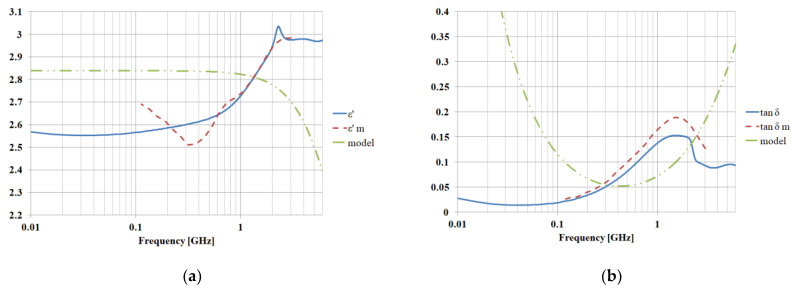
Extending the measurement frequency range through simulations (800 nm Fe simulations between 0.01 GHz and 6 GHz); (**a**) dielectric permittivity and (**b**) loss angle tangent.

In Figure 17, we plot the results of simulation in both lowered frequencies to 10 MHz (for low frequency applications related to classical electromagnetic shielding for electromagnetic compatibility applications) and increased frequencies to 6 GHz (for 5G GSM or 5G wireless applications). In such cases, nanocomposites with non-uniform filler distributions can be successful candidates for innovative applications in microwave domain or telecommunications.

## 4. Conclusions

The importance of ab initio simulation of dielectric properties of nanocomposites with non-uniform filler distribution is of great importance in practice, as the preliminary simulations can lead to a better experimental design of technology before effective measurement of dielectric properties of related samples. This is advisable in order to avoid the unnecessary manufacture of multiple samples for test measurement.

On the other hand, our study might be of significant importance for the fabrication of composites with tailored electromagnetic properties, an example being the advanced electromagnetic shielding systems for electronic or automotive applications, for which until recently, a very restricted dimension of particles was imposed in order to achieve the desired electromagnetic properties, also with an imposed very low variation of the particle dimension. As such, restrictions put a great pressure on particle suppliers and lead to very increased prices for such tailored particles. Our study uses a rendering process that, by randomly unifying the particles dimension towards an average volume index V toward an average exterior surface index S, shows that such restrictive conditions are not necessary for most applications. Hence, particles with a larger variation of particle dimensions can be successfully used, making the related composites more competitive regarding their price and technology. On the other hand, the simulation led to a practical procedure for equating quadratic particles with spherical particles in order to properly analyze the important composites with metallic fillers because the commercial software operates with spherical models for particles.

Finally, the purpose was to find a way to associate the dielectric measurements with the results from the computer simulations, which are mainly based on energetic effects in electromagnetic applications. Accordingly, via use of computer simulations in CST Microwave Studio Software, we determined the transmission/reflection parameters (S_11_ and S_21_), and then we used the free-space NRW procedure to compute the dielectric properties for nanocomposites with metallic/ceramic fillers embedded within a polymer matrix. In order to simulate the intrinsic non-uniformity of real composites, the structure is randomly generated with hundreds of individual rectangular fillers converted to an equivalent spherical model.

The simulation results are favorably in line with the results obtained via advanced dielectric measurements for all simulated/measured filler/matrix combinations and dimensions of particles (e.g., Al and Fe, 7% mass ratio, LDPE matrix, 50 nm and 800 nm reference dimension of the particles).

The proposed method can be used in the beginning stages of the design of nano-filled polymer composites, namely before choosing a specific matrix or specific filler. For filler dimension, we can investigate various combinations in order to fulfill requirements for specific electromagnetic applications.

An additional advantage of the method presented in the paper is the extension of the measurement range for dielectric test equipment towards the GHz domain. In such cases, nanocomposites with non-uniform filler distributions can be successful candidates for innovative applications in microwave domain or telecommunications.

## Figures and Tables

**Table 1:** Temperature regime of the injection process.

Cylinder Zone	5	4	3	2	1
Temperature (°C)	210	200	190	180	170

**Table 2:** LDPE dispersion model data list (measurements).

Frequency (MHz)	Eps′	Eps″	Frequency (MHz)	Eps′	Eps″
3000	2.84	0.265	533	2.51	0.229
2520	2.84	0.322	448	2.51	0.196
2120	2.81	0.379	377	2.50	0.163
1790	2.77	0.417	317	2.49	0.121
1500	2.72	0.420	267	2.49	0.0986
1260	2.67	0.406	225	2.48	0.0836
1060	2.62	0.373	189	2.47	0.0741
895	2.58	0.340	159	2.46	0.0545
753	2.55	0.295	134	2.46	0.0567
634	2.53	0.258	113	2.45	0.0464

## Data Availability

The data presented in this study are available on request from the corresponding author.
